# Association of Electronic Health Literacy With Self-Care and Health Outcomes Among Patients With Type 2 Diabetes Mellitus: Cross-Sectional Study

**DOI:** 10.2196/77856

**Published:** 2026-03-09

**Authors:** Phoenix Kit-Han Mo, Alice PS Kong, Luyao Xie, Virginia WY Chan, Joseph TF Lau

**Affiliations:** 1 Centre of Health Behaviours Research, Jockey Club School of Public Health and Primary Care, Prince of Wales Hospital, Chinese University of Hong Kong, 202D, Shatin, N.T., Hong Kong, 999077, China, 852 2252 8765, 852 2145 7489; 2 Department of Medicine and Therapeutics, Chinese University of Hong Kong, Hong Kong, China; 3 Community Mental Health Association, Hong Kong, China; 4 Public Mental Health Center, School of Mental Health, Wenzhou Medical University, Wenzhou, China; 5 Zhejiang Provincial Clinical Research Center for Mental Disorders, The Affiliated Wenzhou Kangning Hospital, Wenzhou Medical University, Wenzhou, China

**Keywords:** eHealth literacy, diabetes mellitus, patients, health outcomes, self-care

## Abstract

**Background:**

Diabetes mellitus (DM) continues to be a critical public health issue in Hong Kong. Although self-care behaviors help promote health among patients with DM, adherence remains suboptimal. More attention should be paid to eHealth literacy with the development of modern technologies.

**Objective:**

This study aims to assess the level of eHealth literacy among patients with DM and examine its association with self-care and health outcomes.

**Methods:**

A cross-sectional study was conducted among patients with type 2 DM from the DM clinic of a public hospital in Hong Kong. Data on eHealth literacy, self-care, self-care self-efficacy, diabetes distress, glycated hemoglobin (HbA_1c_) control, and sociodemographic information were collected. Multivariable regression analyses were performed, adjusting for relevant sociodemographic and medical variables.

**Results:**

Among the 427 patients with DM recruited, around two-thirds (65.1%) were classified as having a high level of eHealth literacy. Compared to those with lower eHealth literacy, participants with higher eHealth literacy demonstrated significantly higher levels of self-care (*P*<.001) and self-care self-efficacy (*P*<.001) and lower levels of diabetes distress (*P*<.001). Higher eHealth literacy was also associated with greater odds of achieving ideal HbA_1c_ control (<7%) in unadjusted analyses (odds ratio 1.90, 95% CI 1.15-2.81); however, this association was not statistically significant after adjustment for sociodemographic and medical covariates (adjusted odds ratio 1.57, 95% CI 0.99-2.52; *P*=.07).

**Conclusions:**

This study evaluated eHealth literacy levels among patients with DM and examined the associations between eHealth literacy and health outcomes (eg, self-care, self-care self-efficacy, diabetes distress, and HbA_1c_ control). Assessing eHealth literacy in patients with DM could be useful in identifying those who are vulnerable to poorer health outcomes. Promoting eHealth literacy among patients with DM may be important.

## Introduction

Globally, there were 537 million people living with diabetes mellitus (DM) in 2021 [1]. In Hong Kong, the prevalence of DM has increased sharply by 35.1% since 1995 [2]. By 2021, an estimated 7.8% of adults in Hong Kong were affected by DM [1]. It is projected that the prevalence of DM will rise to 9.4% in 2030. DM poses a significant burden to the health care system due to its chronic nature.

There is no known cure for DM, but it is highly controllable. Self-care disease management, including having a healthy diet, exercising, medication adherence, and regular glucose monitoring, is an essential element of diabetes care [3]. Self-care behaviors are strongly associated with positive health outcomes, including lowering blood glucose level, reducing insulin resistance, and reducing the risk of cardiovascular disease [3]; however, adherence to self-care remains suboptimal. A study in Hong Kong among older adults with DM reported that 41.4% had unsatisfactory metabolic control (ie, glycated hemoglobin [HbA_1c_] exceeding the threshold of 7%) [4]. It has also been suggested that patients with DM require support and health information to help with disease management, but these needs have not always been met [5-7].

Online platforms are, and will continue to be, an important resource for obtaining health information, as patients with DM are increasingly playing a larger role in managing their own health [8-10]. The widespread use of digital technologies has facilitated access to information previously available primarily through health professionals [11]. Digital health resources can help individuals with chronic diseases share their experiences and gather information and may help them overcome traditional barriers to self-care [12,13]. Moreover, easy access to health resources provided by digital approaches can increase individuals’ knowledge and awareness regarding medical and health issues, thus empowering them to actively participate in their own health care by making informed and well-considered decisions [14].

Health literacy is defined as “the degree to which individuals have the capacity to obtain, process, and understand basic health information and services needed to make appropriate health decisions” [15]. With the advent of digital technologies, the concept of eHealth literacy (also known as digital health literacy) was introduced to extend health literacy in the digital context [16]. The importance of eHealth literacy for patients to properly use online health information and to participate in informed health care decision-making has been increasingly emphasized [17]. Due to the complexity and amount of information available online, it can be challenging for individuals with little experience in searching for online health information to locate the relevant information. In most cases, information retrieved from the internet is not properly understood by all groups of people [18]. There is also evidence that people have difficulty discerning between trustworthy and untrustworthy health information online [19,20]. Poor-quality, incorrect, and confusing health information online could also be harmful to the patients [21]. It is therefore highly important that individuals are able to effectively process and understand medical information available on the internet.

Increasing attention to eHealth literacy has been observed in recent years. For example, studies in various populations, such as older people [22,23], underserved populations [18], or patients with chronic diseases [17,24,25], have suggested that there is, in general, a low level of eHealth literacy among the study samples. Various sociodemographic factors have been shown to affect the prevalence and extent of online health information seeking, including age, gender, education, socioeconomic status, health status, and internet use [26-31]. Research has further shown that higher eHealth literacy is associated with better health outcomes across different populations, such as better subjective health status [32], health-related quality of life [33-35], lower risk of chronic disease [36], healthy behaviors [37,38], and lower psychological distress [39,40].

Several theoretical models, such as the Transactional Model of eHealth Literacy [41] and the Integrative Model of eHealth Use [42], provide frameworks for understanding how eHealth literacy may influence health outcomes. These models suggest that eHealth literacy can directly or indirectly affect health knowledge, patient–provider communication, as well as health outcomes such as proactive health behaviors and health-related quality of life [41,42]. Among patients with diabetes, higher eHealth literacy has been related to better self-care and other health outcomes through multiple psychosocial and behavioral pathways. For example, a study of 453 adults with diabetes in South Korea found that eHealth literacy was positively linked to self-management via self-efficacy and social support [43]. In a Chinese study, eHealth literacy influenced self-care behaviors indirectly through diabetes knowledge and self-efficacy [44]. Additional studies in South Korea and Taiwan also reported that eHealth literacy was associated with health-promoting behaviors and directly influenced self-care behaviors [45,46]. While previous studies have shown that eHealth literacy is associated with health-related behaviors in diabetes, evidence from patients in Hong Kong remains limited.

Given the substantial health needs of individuals with DM, examining their ability to effectively use high-quality digital health resources and make informed decisions to improve their health would be particularly useful. The purposes of this study were to assess the level of eHealth literacy among patients with DM and to examine the association between eHealth literacy and behavioral (self-care), psychosocial (self-care self-efficacy and diabetes distress), and clinical (HbA_1c_ control) outcomes among patients with type 2 DM in Hong Kong.

## Methods

### Study Design and Participants

A cross-sectional study was conducted among patients with DM. Target participants were patients with type 2 DM in Hong Kong who fulfilled the following criteria: (1) diagnosed with type 2 DM; (2) age between 35 and 65 years; (3) ability to speak and understand Cantonese (the primary language used in Hong Kong). Participants who were receiving psychiatric care at the time of the study were excluded.

### Sample Size Calculation

Based on a previous study by Kim et al [45] conducted among patients with type 2 diabetes, which reported a correlation of *r*=0.15 between eHealth literacy and health-promoting behaviors. The sample size was estimated using G*Power version 3.1, assuming a correlation between eHealth literacy and self-care (*r*=0.15), with a 2-sided significance level of α=.05, and 80% statistical power, the minimum required sample size was 346 participants.

### Data Collection

Eligible patients with type 2 DM attending a diabetes management clinic at a public hospital in Hong Kong were invited to participate in this cross-sectional survey using a clinic-based convenience sampling approach. For recruitment, informational materials (eg, posters and leaflets) were distributed to patients visiting the clinic. Trained research assistants approached eligible individuals who were waiting to receive routine follow-up consultations. Prospective participants received a detailed explanation of the study objectives and procedures. Those who expressed interest in joining the study received an information sheet, which contained detailed information about the purposes and logistics of the study. Written informed consent and contact information were obtained from participants who agreed to participate, and interview appointments were arranged on site. Telephone surveys were administered by trained research assistants within 1 week of the participants’ clinic visit. Participants received a gift coupon valued at HKD 50 (~US $6.5) as a token of appreciation for the time they spent on the study.

### Ethical Considerations

Ethical approval was obtained from the Joint Chinese University of Hong Kong–New Territories East Cluster Clinical Research Ethics Committee (reference number T6080016). Written informed consent was obtained from all participants prior to data collection. Participant information was deidentified to ensure privacy and confidentiality, and all data were handled in accordance with institutional ethical guidelines. No compensation was provided to participants.

### Measures

#### eHealth Literacy

eHealth literacy was measured using the 8-item eHealth Literacy Scale (eHEALS), which was developed to measure individuals’ perceived skills of internet use for health information [47]. Items are rated on a 5-point Likert scale ranging from “1=Strongly disagree” to “5=Strongly agree.” Total scores of the eHEALS range from 8 to 40, with higher scores representing higher levels of eHealth literacy. The scale has been formally translated and validated in Chinese populations [48] and in the Hong Kong context [49-51]. Using the cutoff proposed by Milne et al [52], participants who scored 4 or above on at least 5 out of the 8 eHEALS items were classified as having high level of eHealth literacy. Internal consistency of the scale was satisfactory in the present study (Cronbach α=0.98), which is comparable to that reported in previous studies conducted among general adults (Cronbach α=0.95) [50] and older adults (Cronbach α=0.939) in Hong Kong [51].

#### Level of Self-Care

Self-care was measured by the 15-item Summary of Diabetes Self-Care Activity scale [53], a self-report questionnaire assessing diabetes self-management behaviors across domains including general diet, specific diet, foot care, self-monitoring of blood glucose, smoking, and exercise. Participants were asked to indicate the frequency (days per week) they engaged in each of these activities, with higher scores indicating higher levels of self-care. A validated Chinese version was used [54]. Internal consistency of the scale was satisfactory in the present study (Cronbach α=0.70).

#### Self-Care Self-Efficacy

Self-care self-efficacy was measured using the 20-item Chinese Diabetes Empowerment Scale [55]. Items were adapted and translated from the original 37-item Diabetes Empowerment Scale [56]. The Chinese Diabetes Empowerment Scale measures the degree of psychosocial self-efficacy in carrying out diabetes care in 5 domains: achieving goals, overcoming barriers, coping, suitable methods, and obtaining support. Participants were asked to rate their level of agreement on their perceived ability to handle diabetes-related psychosocial problems on a 5-point Likert scale, from “1=Strongly disagree” to “5=Strongly agree,” with higher scores indicating a greater level of self-care self-efficacy. The internal consistency of the scale was satisfactory in the present study (Cronbach α=0.94).

#### Diabetes Distress

Diabetes-related distress was assessed using the 15-item Chinese Diabetes Distress Scale [57], which measures emotional burden distress, regimen- and social support-related distress, and physician-related distress. Participants were asked to rate the degree to which each item may have distressed or bothered them in the past month on a 6-point Likert Scale from “1=Not a problem” to “6=A very serious problem,” with higher scores indicating a higher level of diabetes distress. The internal consistency of the scale was satisfactory in the present study (Cronbach α=0.93).

#### Sociodemographic Characteristics

Sociodemographic characteristics, including gender, age, education level, relationship status, employment status, monthly income, were obtained. Participants were also asked to report the average time spent on the internet per week. Medical information, such as duration of diagnosis, number of complications, types of treatment, and HbA_1c_ control, was retrieved from participants’ medical records.

### Data Analysis

Descriptive statistics were used to summarize participants’ sociodemographic and medical characteristics and levels of eHealth literacy. Correlations between participants’ sociodemographic and background characteristics, eHealth literacy, and outcome variables were also examined. To evaluate the association between eHealth literacy and outcome variables, participants were classified into high and low eHealth literacy groups based on the cutoff proposed by Milne et al [52]. Independent-sample *t* tests were used to examine the differences in self-care, self-care self-efficacy, and diabetes distress between groups. One-way analysis of covariance (ANCOVA) was also conducted with covariates being adjusted for in each analysis. Sociodemographic and medical variables that were significantly correlated with the respective outcome variables would be included as covariates. To evaluate the association between eHealth literacy and HbA_1c_ control, participants with HbA_1c_ less than 7% were first defined as having ideal HbA_1c_ control. Univariate logistic regression was conducted for obtaining odds ratio (OR) of eHealth literacy on health-related outcomes. Adjusted logistic regression, adjusting for significant sociodemographic and medical variables, was also conducted; adjusted OR for eHealth literacy was then derived. All analyses were performed using SPSS version 21 (IBM Corp). Statistical significance was defined as *P*<.05.

## Results

### Participant Characteristics

A total of 427 patients with type 2 DM completed the survey. The achieved sample size was deemed sufficient for the planned analyses. Slightly more than half of the participants (223/427, 52.1%) were male, and their mean age was 56.53 (SD 7.05) years. About half of them (218/427, 51%) had received a senior secondary level of education or above. The majority of participants (364/427, 85.2%) were married, and more than half of them (240/427, 56.2%) were unemployed. The mean duration of DM diagnosis was 10.69 (SD 8.14) years. Slightly less than two-thirds (261/427, 61.2%) of them had at least one DM-related complication. One hundred ninety out of 427 (44.5%) and 185 out of 427 (43.3%) participants were on oral treatment and on both oral treatment and injections, respectively. Around two-thirds (278/427, 65.1%) of the participants were classified as having high levels of eHealth literacy (Table 1).

**Table 1.** Sociodemographic and medical characteristics of participants (N=427).

Characteristic	Sample
Sociodemographic characteristics	
Gender, n (%)	
Male	221 (52.1)
Female	203 (47.9)
Age (y), mean (SD)	56.53 (7.05)
Education level, n (%)	
Primary or below	113 (26.5)
Junior secondary (Year 7 to 9)	96 (22.5)
Senior secondary (Year 10 to 13)	166 (39)
College or university	51 (12)
Relationship status, n (%)	
Single	32 (7.5)
Married	364 (85.2)
Separated/divorced/widowed	29 (6.8)
Others	2 (0.5)
Employment, n (%)	
Employed	240 (56.2)
Housewife	66 (15.5)
Unemployed	15 (3.5)
Retired	102 (23.9)
Others	4 (0.9)
Monthly income (HKD)^a^, n (%)	
≤9999	68 (16.5)
10,000-19,999	159 (38.7)
20,000-29,999	117 (28.5)
≥30,000	67 (16.3)
Medical characteristics	
Duration of diagnosis (y), mean (SD)	10.69 (8.14)
Number of complications, n (%)	
None	165 (38.8)
1	162 (38.1)
2	78 (18.4)
≥3	20 (4.7)
Types of treatment, n (%)	
No prescribed medication	21 (4.9)
Oral only	190 (44.5)
Injection only	27 (6.3)
Both oral and injection	185 (43.3)
Others	4 (0.9)
Internet-related variables	
Time spent on the internet per week, n (%)	
0 h	64 (15)
<1 h	178 (41.6)
Between 1 and 2 h	121 (28.3)
>2 h	64 (14.9)
eHEALS^b^ score, mean (SD)	23.68 (11.61)
Level of eHealth literacy^c^, n (%)	
Low	149 (34.9)
High	278 (65.1)

a 1 HKD=0.13 USD.

b eHEALS: the 8-item eHealth literacy scale.

c A high level of eHealth literacy was defined as those who scored 4 or above with at least 5 eHEALS items.

### Correlation Between Variables

The correlation between variables is presented in Table 2. Among sociodemographic and medical variables, male gender (*r*=−0.26, *P*<.01), younger age (*r*=−0.43, *P*<.01), higher educational level (*r*=0.50, *P*<.01), being married (*r*=−0.11, *P*<.01), being employed (*r*=−0.24, *P*<.01), higher monthly income (*r*=0.30, *P*<.01), and shorter duration of diagnosis (*r*=−0.14, *P*<.01) had significant correlation with higher level of eHealth literacy. Higher level of eHealth literacy was also significantly correlated with higher level of self-care (*r*=0.15, *P*<.01) and higher level of self-care self-efficacy (*r*=.32, *P*<.01), as well as lower level of diabetes distress (*r*=−0.11, *P*<.05) and lower level of HbA_1c_ (*r*=−0.11, *P*<.05).

**Table 2.** Correlation between variables.

Variable	1	2	3	4	5	6	7	8	9	10	11	12	13	14
Gender^a^	—													
Age	0.12^b^	—												
Education level	−0.30^c^	−0.43^c^	—											
Relationship status^d^	0.04	0.18^c^	−0.13^c^	—										
Employment status^e^	0.17^c^	0.39^c^	−0.20^c^	0.06	—									
Monthly income	−0.10^b^	−0.29^c^	0.40^c^	−0.09	−0.27^c^	—								
Duration of diagnosis	0.13^b^	0.24^c^	−0.21^c^	0.02	0.16^c^	−0.06	—							
Number of complications	−0.10^b^	0.19^c^	−0.13^c^	0.07	0.05	−0.10^b^	0.24^c^	—						
Type of treatment	0.04	0.06	−0.06	−0.01	0.10^b^	−0.04	0.34^c^	0.12^b^	—					
eHealth literacy	−0.26^c^	−0.43^c^	0.50^c^	−0.11^b^	−0.24^c^	0.30^c^	−0.14^c^	−0.09	−0.06	—				
Self-care	0.08	0.01	0.05	−0.10^b^	0.13^c^	0.04	0.10^b^	−0.05	0.09	0.15^c^	—			
Self-care self-efficacy	−0.17^c^	−0.16^c^	0.25^c^	−0.04	−0.05	0.21^c^	−0.05	−0.05	−0.05	0.32^c^	0.30^c^	—		
Diabetes distress	0.07	−0.03	−0.05	0.01	−0.02	-0.20^c^	0.10^b^	0.03	0.02	−0.11^b^	−0.23^c^	−0.46^c^	—	
HbA_1c_^f^	0.08	0.01	−0.11^b^	0.06	0.06	−0.07	0.34^c^	0.18^c^	0.21^c^	−0.11^b^	0.09	−0.10^b^	0.08	—

a Dummy variable was created as 1: male; 2: female.

b *P*<.05.

c *P*<.01.

d Dummy variable was created as 1: married; 2: single/separate/divorced/widowed.

e Dummy variable was created as 1: employed; 2: housewife/unemployed/retired.

f HbA_1c_: glycated hemoglobin.

### Group Difference in Self-Care, Self-Care Self-Efficacy, and Diabetes Distress

Results of independent-samples *t* tests showed that participants classified as having a high level of eHealth literacy had significantly higher levels of self-care (*P*<.001) and self-care self-efficacy (*P*<.001), as well as significantly lower levels of diabetes distress (*P*<.001).

Results of the ANCOVA showed that such differences remained statistically significant after controlling for significant sociodemographic and medical variables. The adjusted mean differences between the high and low eHealth literacy groups were 0.442 for self-care, 3.875 for self-care self-efficacy, and −0.237 for diabetes distress (all *P*<.001) (Table 3).

**Table 3.** Independent sample *t* test and analysis of covariance analyses of group difference in self-care, self-care self-efficacy, and diabetes distress.

Variable	Low eHealth literacy (n=149), mean (SD)	High eHealth literacy (n=278), mean (SD)	Low eHealth literacy (n=149), adjusted mean (95% CI)	High eHealth literacy (n=278), adjusted mean (95% CI)	Mean difference
Self-care	1.78 (1.03)	2.07 (1.16)^a^	1.71 (1.56–1.87)^b^	2.16 (2.02–2.23)^b^	0.442^c^
Self-care self-efficacy	72.46 (7.87)	77.08 (4.51)^c^	73.37 (72.37‐74.37)^d^	77.25 (76.45‐78.05)^d^	3.875^c^
Diabetes distress	2.74 (0.56)	2.44 (0.47)^c^	2.68 (2.60‐2.76)^e^	2.44 (2.38‐2.50)^e^	−0.237^c^

a *P*<.01.

b Adjusted for relationship status, employment, duration of diagnosis, and types of treatment.

c *P*<.001.

d Adjusted for sex, age, education level, and monthly family income.

e Adjusted for monthly family income and duration of diagnosis.

### Logistic Regression on Ideal HbA_1c_ Control

There were 38.7% (96/248) and 26.0% (38/146) ideal HbA_1c_ in participants with low level and high level of eHealth literacy, respectively. Results from the univariate logistic regression analysis showed that participants with a high level of eHealth literacy were more likely to report ideal HbA_1c_ control (unadjusted OR 1.90, 95% CI 1.15-2.81*; P*<.01). After adjusting for significant sociodemographic and medical information, the association was not statistically significant (adjusted OR 1.57, 95% CI 0.99-2.52, *P*=0.070).

## Discussion

### Main Findings and Implications

DM is a chronic disease that requires good knowledge and self-management skills to optimize glycemic control and health outcomes. As digital health technologies are now widely used to support patients, eHealth literacy is of great importance because seeking online health information reflects a complex issue involving information awareness, consciousness, reasoning, and knowledge. It is important to assess patients with DM eHealth literacy, as health care professionals should have a good understanding of the patients’ ability to use electronic resources before recommending the use of online health information [47].

Few studies in Hong Kong have reported the eHealth literacy levels of the population, especially in patients. Several local studies reported mean eHEALS scores of 26.10 (SD 7.70) in 1501 general adults [50] and 27.34 (SD 6.44) among 306 adults aged 60 years or older [51]. This study expands the evidence base among patients in Hong Kong and shows that the mean eHEALS score among 427 patients with type 2 DM was 23.68 (SD 11.61). In addition, using the cutoff proposed by Milne et al [52], approximately two-thirds (278/427, 65.1%) of the patients with DM were considered as having a high level of eHealth literacy. Compared with other studies using the same cutoff, the proportion of patients having a high level of eHealth literacy in the present study was significantly higher than that reported among lung cancer survivors (33.7%) in Canada [52]. This difference may be partly attributable to age, as participants in the present study were younger (mean age 56.5, SD 7.05 y) than those in Milne et al’s study (median age 71 y). This interpretation is further supported by the negative association between age and eHealth literacy observed in the present study and reported consistently in the literature [11,31]. The patients with DM in the present study were likely confident in using the internet for health information and advice; they also demonstrated reasonable skills and self-efficacy required to achieve maximum benefit from the use.

In addition to age, the present study also revealed that among the sociodemographic and medical variables, male gender, higher level of education, currently married, employed, higher monthly income, and shorter duration of diagnosis were associated with higher eHealth literacy. These findings are consistent with the literature showing that eHealth literacy is generally lower among individuals with lower socioeconomic status [14,58-60]. Because the eHEALS was designed to measure an individual’s confidence in their ability to locate and access online health information, it is conceivable that individuals with better socioeconomic status would possess higher ability in understanding and discerning the quality of the health information available on the internet. Individuals who were married and employed may also be more likely to receive a higher level of social support in searching for health information, including those from the online sources.

Understanding the association between eHealth literacy and health-related outcomes is essential for developing effective health communication strategies in the future. It has been found that the use of online health information and eHealth literacy might affect health behaviors [37,45,61,62]. In the present study, higher eHealth literacy was significantly associated with higher levels of self-care and self-care self-efficacy and with lower levels of diabetes distress after controlling for significant sociodemographic and medical variables. These findings are consistent with recent evidence showing that eHealth literacy significantly influences diabetes self-care behaviors among patients with type 2 DM in Saudi Arabia [63]. Although this study also revealed eHealth literacy was significantly associated with better glycemic outcomes in their population [63], the present study did not find a statistically significant association between eHealth literacy and HbA_1c_ among patients with diabetes in Hong Kong after adjustment for covariates. Patients with higher eHealth literacy are not only more inclined to use the internet for health-related information but also are able to understand, evaluate the information obtained, and use quality information to make informed decisions about health. They may therefore be more motivated to adopt healthier lifestyles based on the information that they obtain online [38], more directly leading to better behavioral and psychosocial outcomes. The integrated Model of eHealth Use model also supports the motivational pathways through which eHealth literacy may influence health behavior [42]. Individuals with a higher ability to understand online health information may also be more likely to access health care and interact with health care professionals, therefore more likely to benefit from their health care utilization. In the conceptual model proposed by Paasche-Orlov and Wolf [64], self-efficacy is considered an important mediator linking health literacy and health outcomes. In digital health contexts, self-efficacy has also been found to be a significant mediator in the relationship between eHealth literacy and health outcomes in patients with DM [43,44]. Individuals with higher eHealth literacy are likely to have greater confidence in their ability to effectively utilize online health resources and engage in self-care behaviors, which in turn, is associated with overall behavioral and mental health in patients with DM. However, HbA_1c_ is a more distal biomedical indicator and reflects average glycemic control over the preceding two to three months, which may be less directly associated with eHealth literacy. Future studies are warranted to further elucidate the pathways linking eHealth literacy to glycemic outcomes in this population. Findings of this study suggest that eHealth literacy is an important concept associated with the health of patients with DM. Improving eHealth literacy of patients with DM would potentially be an effective means in improving the health status of the population.

### Implications for Practice

The eHEALS is currently one of the most common tools designed to measure an individual’s confidence in their ability to locate and access online health information [65,66]. It demonstrated high internal consistency and predictive validity in Chinese chronic patients [67], and is associated with a range of health-related outcomes among various populations [22,30,67,68]. Findings of the present study further confirmed its utility in Chinese patients with DM and suggested that it could be an effective screening tool for screening patients’ level of eHealth literacy in clinical settings.

The higher levels of self-care and self-care self-efficacy, and lower levels of diabetes distress documented among participants with higher eHealth literacy also provided important insights that promoting eHealth literacy can have the potential to support patients with DM with self-management behaviors and may result in better DM outcomes. Recent studies have shown that patients with DM who used DM apps had higher eHealth literacy than nonusers and perceived the app to have a greater effectiveness on their health behaviors [68-70]. Health care providers should therefore empower patients with DM with the skills in utilizing online resources. Improving eHealth literacy in patients with type 2 diabetes has important practical implications for self-care and health outcomes. Evidence suggests that improving diabetes knowledge and attitudes toward online health information are likely to be effective strategies for enhancing eHealth literacy in this population [71]. However, it is also noted that participant engagement in eHealth technologies might wane over time. Given the chronic nature of DM, interventions to increase eHealth literacy should make use of content and features that can be personalized and adapted to the individual over time to achieve a sustainable positive effect [72]. Continuous support, training, and monitoring by health care providers are essential to help patients acquire and apply accurate health information effectively, ultimately improving self-care and health outcomes.

### Limitations

There are several limitations that should be noted. First, the present study was cross-sectional in nature; therefore, no causality between the variables could be assumed. Second, self-perceived eHealth literacy was measured in the present study as there was a lack of objective, validated measure in eHealth literacy in the current literature. The current literature seems to have revealed mixed findings on the association between subjective and objective measures of eHealth literacy on health-related internet use [25,36]. However, it is important to note that the eHEALS scale is one of the most used validated scales to measure eHealth literacy. This study was also limited by self-selection bias, that those who agreed to take part in the study might have more favorable views towards internet use and thus a higher level of eHealth literacy. Finally, as the present sample of patients with DM was relatively young and recruited from only one public DM clinic, cautions should be taken when generalizing the results to the general DM population in Hong Kong.

### Conclusion

The internet plays an increasingly important part in disease management among patients with DM. Findings of the present study showed that higher level of eHealth literacy was associated with their better self-care, self-care self-efficacy, lower level of diabetes distress, and having ideal HbA_1c_ control. Assessing the level of eHealth literacy of patients with DM could be useful for identifying patients who are more vulnerable to poorer health. Promoting the eHealth literacy of patients with DM could potentially be associated with better health outcomes.

## Abbreviations

ANCOVA: analysis of covariance
DM: diabetes mellitus
eHEALS: the 8-item eHealth Literacy Scale
HbA_1c_: glycated hemoglobin
OR: odds ratio
